# The Role of Programmed Types of Cell Death in Pathogenesis of Heart Failure with Preserved Ejection Fraction

**DOI:** 10.3390/ijms25189921

**Published:** 2024-09-14

**Authors:** Jan Jankowski, Kamil Oskar Kozub, Marcin Kleibert, Katarzyna Camlet, Klaudia Kleibert, Agnieszka Cudnoch-Jędrzejewska

**Affiliations:** 1 1st Chair and Department of Cardiology, Medical University of Warsaw, 02-091 Warsaw, Poland; 2 Chair and Department of Experimental and Clinical Physiology, Laboratory of Centre for Preclinical Research, Medical University of Warsaw, 02-097 Warsaw, Poland; 3 Department of Pediatric Gastroenterology and Nutrition, Medical University of Warsaw, 02-091 Warsaw, Poland

**Keywords:** heart failure, cardiac failure, cell death, apoptosis

## Abstract

Heart failure with preserved ejection fraction (HFpEF) is a condition that develops in the course of many diseases and conditions, and its pathophysiology is still not well understood, but the involvement of programmed types of cell death in the development of this type of heart failure is becoming increasingly certain. In addition, drugs already widely used in clinical practice, with a good safety profile and efficacy demonstrated in large-group clinical trials, seem to be exerting their beneficial effects on cardiovascular health. Perhaps new drugs that reduce the susceptibility of cells to programmed types of cell death are under investigation and may improve the prognosis of patients with HFpEF. In this article, we summarize the current knowledge about the pathogenesis of HFpEF and the role of programmed types of cell death in its development. Additionally, we have described the future directions of research that may lead to the improvement of a patient’s prognosis and potential treatment.

## 1. Introduction

**Figure 1:**
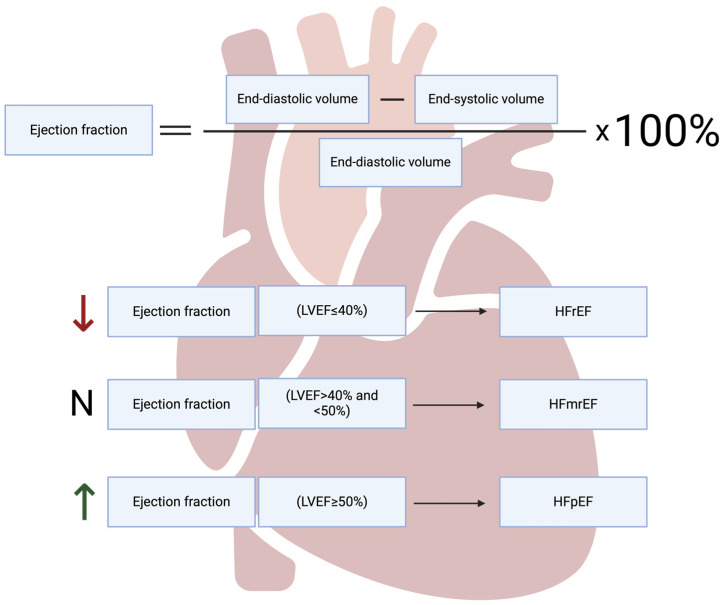
The classification of heart failure based on ejection fraction. LVEF—left ventricle ejection fraction, HFrEF—heart failure with reduced ejection fraction, HFmrEF—heart failure with mildly reduced ejection fraction, HFpEF—heart failure with preserved ejection fraction. Created with Biorender.com.

Heart failure (HF) is a condition in which the heart is unable to maintain a cardiac output commensurate with the body’s needs. HF can be divided according to left ventricular ejection fraction (LVEF) (Figure 1).

In this article, we will focus on heart failure with preserved ejection fraction (HFpEF), which is not as well evaluated as heart failure with reduced ejection fraction (HFrEF). It is characterized by impaired cardiac relaxation and increased passive cardiac stiffness [1].

In this article, we discuss the role of the different types of programmed cell death (PCD) in the development and progression of HFpEF. Additionally, we summarize the knowledge about new, potential therapeutic targets that have been evaluated in pre-clinical research.

**Figure 2:**
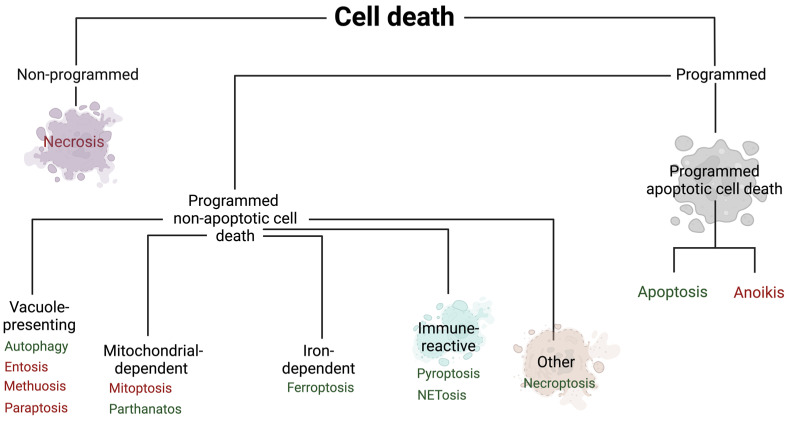
The division of cell death types. Red color—that type of cell death is not described in this article due to the lack of data in HFpEF; green color—that type of cell death is described. Created with Biorender.com.

Cell deaths can be categorized as programmed or non-programmed (non-PCD). PCD is distinguished by strictly regulated mechanisms. In contrast, non-PCD occurs under the influence of accidental cell damage. PCD is further divided into apoptotic and non-apoptotic cell death. Moreover, programmed apoptotic cell death is characterized by maintaining the integrity of the cell membrane, while programmed non-apoptotic cell death usually involves the rupture of the cell membrane. Another difference is the dependence of cell death on caspases, while the first of them is dependent and the second is independent [2] (Figure 2).

## 2. Non-Programmed Cell Death

Necrosis is the process of cell death caused by irreversible cell damage. It is caused by many non-specific factors, for example, hypoxia, high doses of metabolites, etc. Necrosis is not a molecularly regulated process that can be modified by biochemical pathways. It is characterized by a chaotic inactivation of intracellular processes [2]. Therefore we can only influence the processes causing the occurrence of necrosis, e.g., minimizing ischemia. For this reason, we have omitted this topic from our article.

## 3. Programmed Cell Death

### 3.1. Programmed Apoptotic Cell Death

**Figure 3:**
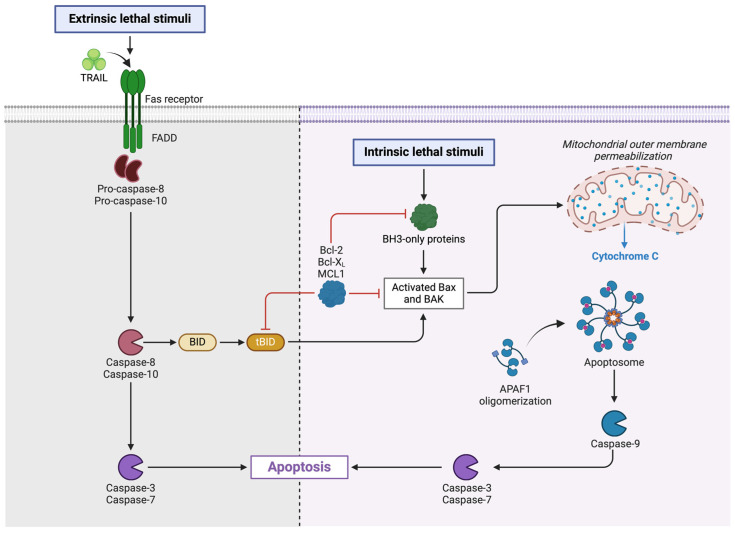
The mechanism of the extrinsic and intrinsic apoptotic pathways. TRAIL—tumor necrosis factor-related apoptosis-inducing ligand, Fas receptor—Fas cell surface death receptor, FADD—Fas-associated death domain, BID—BH3 interacting-domain death agonist, tBID—truncated BID, Bcl-2—B cell lymphoma 2, Bcl-xL—B-cell lymphoma-extra-large, MCL1—myeloid leukemia 1 protein, BH3—Bcl-2 homology domain 3, Bax—Bcl-2-associated X protein, BAK—Bcl-2 antagonist/killer, APAF1—apoptotic protease activating factor 1, Created with Biorender.com.

Programmed apoptotic cell death is characterized by caspase dependence. During it, the integrity of the cell membrane is maintained. It can be divided into apoptosis and anoikis. Both types of cell death have the same pathways. The difference lies in the initiation of the process. Anoikis is listed as a distinct type because it is initiated as a loss of connection between the cell and the extracellular matrix. We are not focusing on it in this article due to a lack of data in the context of HFpEF. In contrast, apoptosis is well-investigated. Two classic signaling pathways to trigger apoptosis (extrinsic and intrinsic) are known (Figure 3). Some authors additionally distinguish the perforin/granzyme pathway [2].

#### 3.1.1. Apoptosis

In all types of HF, there is an increased rate of apoptotic cells (0.08–0.25% as assessed by terminal deoxynucleotidyl transferase dUTP nick-end labeling) compared to healthy hearts (0.001–0.01%) [3]. Moreover, it was shown that even increased ventricular pressure, which was achieved by using banding of the aorta or pulmonary artery, is associated with a higher apoptotic rate [4,5].

The inhibition of apoptosis contributes to the preservation of cardiac function, as was summarized in the well-written article [3]. However, because it is an extensive topic, in our article, we have chosen the most prominent substances, which will be described in further parts of this article. On the other hand, the inhibition of apoptosis can contribute to the abnormal overgrowth of the myocardial matrix and fibrosis because of the proliferation of apoptosis-resistant fibroblasts [6].

It is worth paying attention to the cellular pathways that determine the course of apoptosis. The apoptosis can be activated through three different pathways. The most important of them are intrinsic and extrinsic. The intrinsic pathway is activated by intracellular stress by the release of cytochrome c. This is dependent on the balance between anti-apoptotic (B cell lymphoma 2 [Bcl-2], B-cell lymphoma-extra-large [Bcl-xL]) and pro-apoptotic (Bcl-2-associated X protein [Bax], Bcl-2 homology domain 3 [BH3]) proteins. The extrinsic pathway involves the activation of death receptors with corresponding external ligands to form intracellular complexes. Both pathways lead to the activation of the cascade of the corresponding caspases and cell death [7].

##### Apoptosis Plays a Role in HF and HFpEF

The role of apoptosis in the development of HF was directly demonstrated by Moorjani et al. [8]. They studied apoptotic gene expression in left ventricular tissue in patients with different stages of development of HF caused by volume overload. The significantly lowest expression of pro-apoptotic factors: Bcl-2-associated X protein (Bax), p53, tumor necrosis factor receptor 1 (TNFR1), and caspases 3, 8, and 9 and anti-apoptotic mitochondrial factor—Bcl-xL was observed in the group of patients with preserved left ventricular function and increased with progression to HF. However, there was an increase, but not statistically significant, for BCL2 associated agonist of cell death (Bad), Bcl-2 (genetic regulators of mitochondria), or Fas cell surface death receptor (Fas) expression and DNA fragmentation between the groups. This shows an effect of volume overload on the increase in apoptosis regulated by intrinsic and extrinsic pathways in HF. An increase in the anti-apoptotic protein Bcl-xL correlated with worsening left ventricular function. This suggests the activation of compensatory mechanisms that inhibit apoptosis.

**Figure 4:**
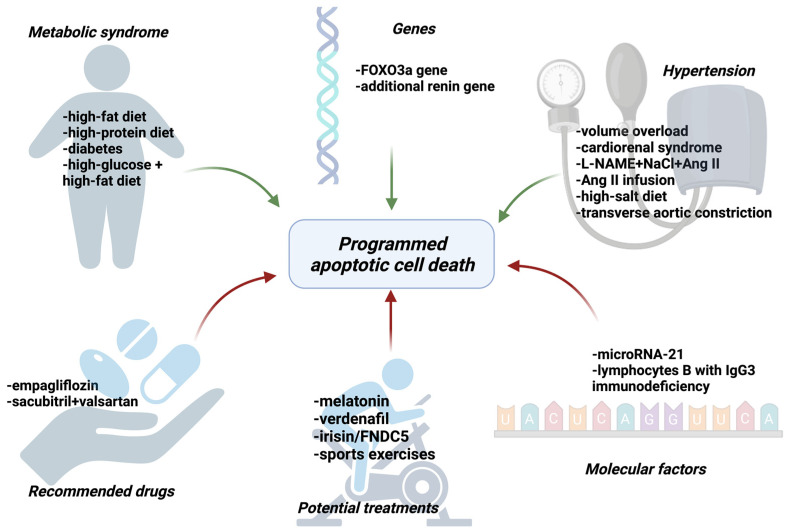
The factors that activate (green arrows) or inhibit (red arrows) apoptosis of cardiomyocytes among patients with HFpEF. FOXO3a—Forkhead box O3a, L-NAME—Nω-nitrol-arginine methyl ester, NaCl—sodium chloride, AngII—angiotensin II, FNDC5—Fibronectin type III domain-containing protein 5, microRNA—micro ribonucleic acid, IgG—immunoglobulin G. Created with Biorender.com.

There are many studies indicating the influence of apoptosis on the development of HFpEF which will be discussed in our article [9,10,11,12,13,14,15,16,17,18,19,20,21,22,23,24,25,26,27]. The most important factors regulating apoptosis are presented in Figure 4.

Interesting results are provided by TNF-related apoptosis-inducing ligand (TRAIL) studies. TRAIL receptors are in the cell membrane. TRAIL-Receptor 1 and TRAIL-Receptor 2 are death receptors. They are responsible for the induction of apoptosis by the extrinsic pathway. TRAIL-Receptor 2 is significantly increased in HFpEF in comparison to healthy patients. TRAIL-Receptor 2 was also significantly increased in obese versus lean patients with HFpEF [9]. These results suggest the influence of TRAIL pathway activity on the development of HFpEF. However, higher TRAIL levels in HFpEF patients are associated with a better prognosis [10]. It has also been studied that a low TRAIL level in patients with HFrEF is statistically significantly associated with a higher risk of death [28]. However, whether the TRAIL pathway has a protective effect in patients with HFpEF has not yet been clarified. This relationship is not fully understood, seems surprising, and requires further research.

##### Drugs Recommended in HFpEF Reduce Apoptosis

Among the drugs recommended for use in HFpEF [29], only empagliflozin and sacubitril/valsartan have been tested on apoptosis.

Yang et al. [11] studied the effect of early empagliflozin administration (20  mg/kg/day) on an animal model (rat with cardiorenal syndrome caused by 5/6 subtotal nephrectomy and dilated cardiomyopathy by doxorubicin) and cell culture (H9c2 cells—rat cardiomyocytes with para-Creso-induced apoptosis). This animal model may correspond to HFrEF due to the infusion of doxorubicin. However, subtotal nephrectomy is used as a model for HFpEF, and all measured LVEFs were above 50%, so we classified it as an HFpEF model. As markers of apoptosis, cleaved poly(ADP-ribose) polymerase (PARP, plays a key role in repairing DNA breaks; therefore, its caspase cleavage is a marker of apoptosis), cleaved caspase-3 and mitochondrial Bax expression were used. The increase of apoptotic markers was significantly higher in empagliflozin-treated and untreated rats with cardiorenal syndrome compared to healthy controls. However, empagliflozin administration attenuated the negative effect of cardiorenal syndrome on apoptosis. In addition, transthoracic echocardiography (ECHO) showed significant improvement in cardiac function in the empagliflozin group. Among cell cultures, empagliflozin had a protective effect on the occurrence of apoptosis as measured by flow cytometry.

Yeh et al. [12] studied the effect of sacubitril/valsartan on rats with cardiorenal syndrome caused by a high-protein diet and H9c2 cells with H_2_O_2_-induced apoptosis. As markers of apoptosis were also used cleaved PARP, cleaved caspase-3 and mitochondrial Bax expression. Sacubitril/valsartan administration had a similar protective effect on the occurrence of apoptosis as measured by flow cytometry.

These studies had a similar methodology and a beneficial effect of both drugs on apoptosis was observed. In addition, the supply of drugs correlated with both the reduction in apoptosis and the improvement of functional and structural parameters of the heart, including maintenance of normal LVEF values and less myocardial fibrosis.

##### Searching for New Treatment Options with a Potential Target in Apoptosis

Liu et al. [13] studied the effect of melatonin on HFpEF. They used mice on a high-fat diet (HFD) and administered melatonin (50 mg/kg) to them. The significant overproduction of reactive oxygen species (ROS) in HFD compared to mice on a normal diet was observed. Moreover, the melatonin supplementation in the HFD almost completely inhibited ROS production compared to untreated mice. Similarly, they showed that an HFD is related to increased apoptosis, which was reduced by administration of melatonin. It was confirmed by the increased expression of Bcl-2 in the group of mice treated with melatonin. This indicates a possible positive effect of melatonin administration in obese patients with HFpEF.

Patients with HFpEF have been reported to have lower myocardial cyclic guanosine monophosphate (cGMP) levels in comparison to the control group [30], whereas phosphodiesterase-5A inhibition increases cGMP levels. Matyas et al. [14] studied the effect of vardenafil (phosphodiesterase-5A inhibitor) on the Zucker diabetic fatty rats, a rodent model of HFpEF. Animals received a placebo or vardenafil (10 mg/kg). Increased apoptosis assessed by terminal deoxynucleotidyl transferase dUTP nick end labeling (TUNEL) and cleaved caspase-3 was observed in Zucker diabetic fatty rats compared to the normal diet group. Vardenafil significantly decreased the number of TUNEL-positive nuclei, but cleaved caspase-3 was not significantly different. Vardenafil treatment was also associated with maintained diastolic function which reduced the risk of HFpEF development. In the group of HFpEF patients with diabetes, vardenafil may also provide potential benefits.

Lin et al. [15] studied db/db mice (an animal model of HFpEF and diabetic cardiomyopathy) with adenovirus encoding fibronectin type III domain containing 5 (FNDC5) protein or treated with recombinant human irisin (a cleaved form of FNDC5) with a non-diabetic control group (db/+). Db/db mice had significantly increased levels of cleaved caspase-3 (protein, marker of apoptosis) and a decreased Bcl-2/Bax ratio compared to the control group. In addition, FNDC5 overexpression and irisin supplementation attenuated diastolic dysfunction and cardiac structural remodeling in db/db mice compared to the control group. In vitro studies have shown that FNDC5 prevents apoptosis in high glucose and high fat-treated cardiomyocytes; however, it is not a direct model of HFpEF. Irisin is a novel myokine and it is associated with the regulation of energy metabolism. In another study, it was proved that irisin has a protective effect on cardiovascular diseases. In hypertrophic hearts expression of it is elevated. Irisin administration reduces cardiomyocyte hypertrophy induced by in vitro angiotensin II administration. In contrast, its knockdown exacerbates hypertrophy [31]. This is another treatment that may have application in the management of patients with HFpEF and diabetes.

Wang et al. [16] investigated the effect of exercise in db/db mice on heart function and apoptosis. The TUNEL assay showed increased apoptosis. Also, decreasing the Bcl-2/Bax ratio in db/db mice compared to wild-type mice was observed which indicated increased apoptosis. This ratio was significantly reversed in exercised db/db mice compared to sedentary db/db mice. Exercise in groups of wild-type mice had no effect on apoptosis. This suggests a beneficial effect of exercise on preserving heart function in diabetes-induced HFpEF. We know the cardioprotective effect of exercise. We also know that exercise inhibits the apoptotic loss of cardiomyocytes in the aging heart [32]. Many molecular pathways responsible for this have already been discovered, but the mechanisms involved are not fully understood.

These studies show that some unused therapies in HFpEF may have a positive effect on reducing apoptosis. Among them are vardenafil, melatonin, irisin/FNDC5, and physical activity. Further studies on these agents or their derivatives are needed to verify whether they can be used to treat patients with HFpEF (Table 1).

##### Other Targets of Drugs (Genes or Proteins)

More and more studies are being conducted on HFpEF models, testing the influence of subsequent genes or proteins on apoptosis. Inhibiting or stimulating these factors may be a potential target for new drugs.

Tao et al. [33] studied the effect of micro ribonucleic acid 21 (miR-21) expression in vitro in H9C2 cells stimulated with high glucose and palmitate. MiR-21 is one of the regulators of inflammatory processes and cell death, and it is highly expressed in fibroblasts and cardiomyocytes. The authors showed an increased level of miR-21 in stimulated cells in comparison to the control, which was negatively correlated with cell apoptosis. Also, they confirmed that miR-21 knockout can promote apoptosis. Furthermore, Dai et al. [17] also studied the expression of miR-21 but on animal models (db/db mice) instead of cell culture. They confirmed that diabetes is associated with reduced levels of miR-21. Also, mice with HFD-induced diabetes compared to control had reduced miR-21 levels. Another research team led by Dong et al. [18] showed that the expression of the abovementioned miRNA is higher in cardiac fibroblasts than in myocardiocytes. Recall that excessive growth of fibroblasts contributes to cardiac fibrosis. After in vitro fibroblast transfection of pre-microRNA-21 cell cultures, the expression of Bcl-2 (anti-apoptotic gene, intrinsic pathway) increased at the RNA level. A rat model of HFpEF injected with a miR-21 antagonist was further compared with appropriate controls. They used male Sprague Dawley (SD) rats with transverse aortic constriction or sham-operated as a model of HFpEF. Although this model raises doubts as to whether it is appropriate for HFpEF, the researchers showed with the ECHO that the diastolic functions of the transverse aortic constriction group were impaired. Three weeks after injection of the miR-21 antagonist, expression of Bcl-2 in the cardiac fibroblasts was reduced. Injection of a miR-21 antagonist also reduced cardiac atrophy and cardiac fibrosis. The study suggests that miR-21 affects the expression of the anti-apoptotic Bcl-2 gene, thereby inhibiting fibroblasts apoptosis. This suggests an effect of miR-21 on promoting the development of HFpEF. Studies on the effect of miR-21 in a model of diabetic cardiomyopathy leading to HFpEF are divergent. There are reports that miR-21 contributes to the inhibition of cardiomyocyte apoptosis and improves diastolic function [17,33]. On the other hand, other researchers have confirmed that miR-21 also inhibits fibroblast apoptosis, which results in cardiac fibrosis and progressive diastolic dysfunction [18]. In light of these divergent reports, further research is needed to clarify the role of miR-21. However, if it were possible to selectively inhibit cardiomyocyte apoptosis or selectively stimulate fibroblast apoptosis, then miR-21 seems to be an interesting therapeutic target.

Chaanine et al. [19] injected cardiotropic adeno-associated virus, which transmitted the dominant-negative transcription factor forkhead box O3a (FOXO3a) gene to SD rats with ascending aortic banding. They found that FOXO3a upregulates mRNA expression of the mitochondrial apoptotic marker Puma (BH3 protein family, intrinsic pathway) in adult cardiomyocytes. The delivery of this transcription factor improved cardiac function, mitochondrial ultrastructure and function as well as diastolic function. However, we have to remember that even if this animal model is often used as a model of HFpEF, it can cause both diastolic and systolic insufficiency which should be taken under consideration during results interpretation. The FOXO3a-BNIP3 (BNIP3—BCL2/adenovirus E1B 19-kDa protein-interacting protein 3) pathway has previously been shown to modulate mitochondrial function and influence apoptosis in rodent models of HF. Chaanine et al. [20], in another study, examined the expression of this pathway in human heart muscle biopsies. The total expression of BNIP3 (BH3 protein family, intrinsic pathway) was significantly increased in biopsies from patients with HFpEF compared to normal. However, the expression of this protein was non-significantly higher in HFrEF compared to HFpEF. This confirms studies that HF is associated with increased activation of the FOXO3a-BNIP3 pathway. However, it seems to play a more important role in reduced LVEF.

Increased expression of angiotensin II is observed in patients with diabetic cardiomyopathy. Therefore, Connelly et al. [21] investigated the effect of diabetes and renin overexpression on apoptosis in animal models of HFpEF (SD (mRen-2)27 transgenic rats). It is a transgenic rat with the additional renin gene that develops diabetic nephropathy, whereas exposure to streptozocin causes diabetes in these rats. The animals were randomized and divided into two groups: with and without induced diabetes. They were compared with non-transgenic diabetic rats and normal rats. Assessment of rodent hearts under an electron microscope revealed normal testicular morphology in non-diabetic rats and dense nuclear chromatin in the nuclear periphery, suggesting apoptosis in the study group. Also, apoptosis was noted greater extent in people with diabetes with additional renin gene rats. These findings suggest that diabetes and higher renin expression contribute to HFpEF, which may manifest as increased apoptosis. Additionally, this animal model may become an experimental model to study pathomechanisms and new drugs in diabetic cardiomyopathy.

The role of immune mechanisms in HF is poorly understood. Cordero-Reyes et al. [22] investigated the role of B lymphocytes in its progression. They investigated a model of non-ischemic cardiomyopathy induced by the use of Nω-nitrol-arginine methyl ester (L-NAME, non-selective inhibitor of nitric oxide synthase, used experimentally to induce hypertension) and sodium chloride (NaCl) in drinking water and the infusion of angiotensin-II in mice for 35 days. The LVEF of the animals varied (49.4 ± 7%). The study groups had induced immunodeficiency by the depletion of B and T lymphocytes. The study showed a non-significant positive correlation between the occurrence of apoptosis and the presence of immunoglobulin G3 (IgG3). Apoptosis was assessed by the occurrence of the Bax. Also, the absence of B cells in this HF model resulted in less hypertrophy, collagen deposition, and preservation of left ventricular function. This study suggests that the full activation of apoptotic pathways in this model of hypertension-induced left ventricular dysfunction is mediated by B lymphocytes.

The above studies show the association of FOXO3a and renin genes with increased apoptosis in animal models of HFpEF. On the other hand, the depletion of B lymphocytes producing IgG3 antibodies has been shown to reduce apoptosis. In turn, the role of miR-21 expression is ambiguous. It seems reasonable to search for drugs that act on these factors. Therefore, further research is needed on further genes or molecular pathways that may affect apoptosis in HFpEF models.

##### Metabolic Syndrome Enhances Apoptosis

Many of the above studies are based on animal or cell models to imitate diastolic HF induced by diabetes [14,15,16,17,21] or an HFD [13]. These studies clearly indicate increased apoptosis of cardiomyocytes. Notably, in diabetic patients, cardiomyocyte apoptosis may be increased 85-fold and fibroblast apoptosis 26-fold in left ventricle biopsy tissue [23]. Therefore, it seems clear that a high-sugar diet and an HFD have a negative effect on heart function. This indicates the importance of limiting dietary sugars and fats in patients with HFpEF to protect cardiomyocytes from PCD. It also seems important to control comorbidities such as diabetes mellitus or hyperlipidemia.

##### Hypertension Enhances Apoptosis

Some of the above studies also indicate the influence of hypertension on increased apoptosis [18,19,22]. There are also many other studies showing this effect.

Zhou et al. [24] compared factors found in HFpEF and hypertension using functional enrichment analysis of overlapping network modules. Among the main biological processes linking both clinical conditions was apoptosis (12.82%).

An effective method of imitating HFpEF in mice is a continuous infusion of low-dose angiotensin II (0.2 mg/kg·day) [34]. This model is widely used due to its low cost and availability. Cappetta et al. [25] noted significantly higher activation of apoptosis using the TUNEL test after 14 days of angiotensin II continuous infusion (1.5 mg/kg·day) in comparison to mice without mini-pump implantation. Moreover, these authors showed that the expression of Bax was non-significantly higher in the study group. This study also investigated glucocorticoid-induced leucine zipper (a gene involved in cell proliferation, survival, and inflammatory signaling) knockout. However, apoptosis occurred at the same frequency in mice with and without glucocorticoid-induced leucine zipper knockout.

Brandt et al. [26] performed the histological analysis of left ventricle apex tissue using TUNEL and activated caspase 3. It showed the non-significant pro-apoptotic effect of a high-salt diet in animal model HFpEF with hypertension. Similarly, Zhang et al. [27] studied the effect of a high-salt diet in Dahl salt-sensitive rats which is a well-known HFpEF model. However, in this research, a significantly higher level of apoptosis was observed in the study group compared to the low-salt diet. A real-time polymerase chain reaction (RT-PCR) analysis showed that Bcl2l14 and Bax (pro-apoptotic factors) were upregulated, and Bcl2l10 (anti-apoptotic factor) was significantly downregulated in high salt diabetic rats. This indicates enhanced cardiomyocyte apoptosis in rats fed a high-salt diet that imitates hypertension.

Thus, it seems that hypertension is an important factor in increasing cardiomyocyte apoptosis. This only confirms that, as has long been known, maintaining proper blood pressure is crucial in patients with HF.

### 3.2. Programmed Non-Apoptotic Cell Death

#### 3.2.1. Ferroptosis

Further research is required to comprehend the exact mechanisms responsible for the initiation and execution of ferroptosis. However, it is evident that ferroptosis is linked to iron metabolism and lipid oxidation [2,35]. Ferroptosis is caused by the accumulation of ROS that are produced during iron-dependent lipid peroxidation.

The process of ferroptosis involves alternations in mitochondrial morphology. The observed changes include a reduction in mitochondrial volume, an increase in bilayer membrane density, and the disappearance of mitochondrial cristae. There are no changes in the structure of the cell nucleus or cell membrane, which are characteristic of apoptosis [35,36].

Many pre-clinical and clinical studies have shown that antidiabetic drugs known as sodium-glucose cotransporter 2 (SGLT2) inhibitors provide cardiovascular benefits [12]. Ma et al. [37] investigated the effect of canagliflozin treatment on a rat model of HFpEF induced by a high-salt diet. Transmission electron microscopy (TEM) images of heart samples revealed changes indicative of ferroptotic cell death, such as mitochondria with thickened membranes and reduced cristae. However, these changes appeared to be less pronounced in the group that received canagliflozin. ECHO also demonstrated an improvement in left ventricular diastolic function and a reduction in myocardial hypertrophy in the treated group compared to the untreated group. The proteome was analyzed and compared among the treated, untreated, and control groups. The proteins whose expression varied significantly between the groups were mainly associated with lipid metabolism, ion metabolism, sulfur compound metabolism, and regulation of cellular protein localization. All of these were integrated within the ferroptosis metabolic pathway. The analysis revealed its higher concentrations in rats HFpEF compared to the control group. These indexes were significantly reduced in the rats treated with canagliflozin. The authors demonstrate the association between myocardial cell ferroptosis and the development of HFpEF. They hypothesize that a key mechanism behind the efficacy of including SGLT2 inhibitors in the treatment of this condition is the reduction of this pathological process.

Additionally, Kitakata et al. [38] demonstrated the significant benefits of imeglimin, a potential new treatment for type 2 diabetes, in a mouse model of HFpEF induced by an HFD and L-NAME. The study included three groups of mice: a control group on a normal diet, a group on an HFD and L-NAME, and a group on an HFD and L-NAME treated with imeglimin from week 10. The mice in the HFpEF group showed increased body weight, visceral fat mass, and impaired glucose tolerance, as well as cardiac hypertrophy and fibrosis. Imeglimin treatment led to a significant reduction in body weight and visceral fat mass and improved glucose tolerance. In addition, a decrease in cardiac hypertrophy and fibrosis, as well as an improvement in left ventricular diastolic function, was observed. Exposure to HFD + L-NAME resulted in a reduction in the cardiac expression of glutathione peroxidase 4 (GPX4), a regulator of mitochondrial redox balance and ferroptosis. Imeglimin reinstated the expression of GPX4 in mice exposed to HFD + L-NAME. Therefore, this study provides evidence for the potential importance of regulating the ferroptotic cell death process in the positive effect of imeglimin on a model of HFpEF.

Zhang et al. [39] demonstrated that levosimendan, a drug used for the treatment of HF with an inotropic and vasodilatory effect, exerted a protective effect against ferroptosis in myocardial tissue from HFpEF mice. This was evidenced by an increase in the glutathione/glutathione disulfide ratio, upregulation of key ferroptosis regulators such as GPX4, cystine/glutamate antiporter SLC7A11 (xCT), and ferroptosis suppressor protein 1 (FSP-1), and a reduction in intracellular ferrous ion, malondialdehyde, and 4-hydroxynonenal levels. After four weeks of levosimendan treatment, there was a significant improvement in left ventricular diastolic dysfunction, cardiac hypertrophy, pulmonary congestion, and exercise exhaustion. It is worth noting that levosimendan had a positive effect on junction proteins involved in maintaining endothelial barrier integrity and cardiac myocyte connectivity. Of particular significance was the upregulation of connexin 43, a gap junction channel protein predominantly expressed in cardiomyocytes, which played a crucial role in mediating mitochondrial protection.

There are also other therapeutic targets directed at the factors causing HFpEF. Among them is elabela, which protects against hypertension-induced cardiac microvascular endothelial cell ferroptosis and cardiac remodeling in hypertensive mice [40]. Another key pathogenic factor that initiates diabetic cardiomyopathy through ferroptosis is advanced glycation end-products [41].

On the other hand, inhibition of ferroptosis in fibroblasts leads to cardiac fibrosis. Su et al. [42] conducted a series of experiments to investigate the role of Sirtuin 3 (SIRT3) induced ferroptosis in myofibroblasts and its implications in cardiac fibrosis. SIRT3 is located in the mitochondria and is involved in cellular energy homeostasis and oxidative stress regulation. Furthermore, overexpression of SIRT3 and inhibition of p53 acetylation reverse ferroptosis in cultured myofibroblasts induced by Erastin, a ferroptosis inducer. The study introduced a novel SIRT3 knockout in a mouse model that suppressed cardiac fibrosis. It supports the importance of the SIRT3/acetylated p53 pathway-induced ferroptosis in myofibroblasts as a potential therapeutic target for inhibiting cardiac fibrosis development. The authors validated their results by treating SIRT3-knockout mice with the ferroptosis inhibitor Ferrostatin-1. A reduction in cardiac fibrosis was observed. The study highlighted that SIRT3 deficiency-induced ferroptosis contributes to the development of cardiac fibrosis, shedding light on potential treatments for cardiac dysfunction.

The researchers have also investigated the role of ferroptosis in clinical studies. It was shown that supplementation with coenzyme Q10 may reduce oxidative stress reduction and, consequently, ferroptosis [43]. Adarsh et al. [44] reported the beneficial effects of long-term (14-month) coenzyme Q10 supplementation in patients with hypertrophic cardiomyopathy. The study found that the supplementation improved both quality of life and objective ECHO parameters. Pierce et al. [45] showed the effects of 12 weeks of supplementation with ubiquinol (a reduced form of coenzyme Q10) and D-ribose in 216 HFpEF patients, consistent with the Adarsh et al. [44] study. In contrast, Sobirin et al. [46] and Samuel et al. [47] did not observe significant improvements in cardiac diastolic function or other parameters, but these studies only described the short-term effects.

However, still most of the evidence related to ferroptosis comes from studies on animal models and has to be proved in clinical studies (Table 2).

#### 3.2.2. Autophagy

Autophagy is a cellular process that degrades and recycles damaged or dysfunctional cellular components. It involves the formation of autophagosomes that engulf the targeted material and fuse with lysosomes for degradation. Autophagy is crucial for maintaining cellular homeostasis [2]. The pathophysiological mechanisms of adverse cardiac remodeling are related to impairments of myocardial oxygen supply and demand, increased apoptosis and necrosis, and decreased autophagy. Autophagy is initiated by a lack of nutrients and inhibited by an excess of nutrition. Therefore, nutrient-sensing pathways, such as the 5′AMP-activated protein kinase (AMPK) and mammalian target of rapamycin kinase (mTOR) pathways, regulate this process. Various pharmacotherapeutic agents have been reported to protect diabetic hearts by inducing cardiac autophagy through the activation of AMPK or the suppression of mTOR.

Xu et al. [48] examined autophagy in cells exposed to high glucose concentrations. The 48-h treatment with dapagliflozin was able to reverse the decrease in phosphorylation of AMPK, the increase in phosphorylation of S6 kinase ribosomal protein (a downstream protein of mTOR), and the decrease in autophagic flux caused by high glucose levels. Additional studies showed that both AMPK gene knockout and administration of rapamycin, an inhibitor of the mTOR pathway, blocked the beneficial effects of dapagliflozin. These findings demonstrate that dapagliflozin modulates autophagy by affecting AMPK and mTOR. The study indicates that dapagliflozin could be a potential treatment option for managing conditions related to impaired autophagy, especially in cases of high glucose concentrations, such as HF, in diabetes mellitus patients.

Xie et al. [49] demonstrated that diabetes inhibits autophagy in the myocardium of OVE26 mice, as evidenced by a reduced amount of autophagosomes, lower LC3-II protein levels, and decreased Beclin-1 protein expression. The accumulation of abnormal proteins and damaged organelles, such as mitochondria, may result in cardiac dysfunction. Furthermore, chronic metformin therapy significantly increased autophagic activity and preserved cardiac function in diabetic mice. However, this effect was not observed in mice overexpressing a cardiac-specific dominant negative form of AMPK, which acts as an AMPK inhibitor. Metformin treatment enhances the expression and action of AMPK, which regulates autophagic mechanisms. This may be responsible for potential cardiovascular benefits in diabetic patients.

Sulforaphane, which is found in broccoli sprout powder, and curcumin, which is found in turmeric, are natural molecules present in foods that can restore previously reduced levels of autophagy. Both molecules have been tested in animal models of diabetes and have been shown to have a beneficial effect on the myocardium through the AMPK/mTOR-dependent pathway [50,51].

The regulation of autophagy is not limited to the AMPK/mTOR-dependent pathway. Factor forkhead box O1 (FOXO1) and factor forkhead box O3 (FOXO3) act as transcriptional regulators of autophagy and have potential as therapeutic targets in diabetic cardiomyopathy with autophagy dysregulation.

Trehalose activates FOXO1-mediated autophagy in mice, independent of the Akt/mTOR pathway [52]. The activation of autophagy may offer further therapeutic benefits of trehalose in managing diabetic complications, including diabetic cardiomyopathy.

It is important to note that excessive activation of autophagy, specifically mitophagy, caused by FOXO1/FOXO3a activation in the hearts of diabetic animals leads to the loss of mitochondria and induces cardiac dysfunction in Goto-Kakizaki rats. This indicates that abnormal myocardial remodeling and HF may be caused by the dysregulation of autophagy mechanisms rather than their potentiation or impairment.

#### 3.2.3. Parathantos

Parthanatos is a type of cell death associated with mitochondria. It is characterized by the independence of the process pathways from caspases and increased activation of PARP [2].

Increased activation of PARP-1 was observed in rodents after myocardial infarction [53]. In other experiments with ischemic injury, PARP-1 knockout in mice showed improved myocardial contractility and viability compared to wild-type mice [54,55]. Also, the administration of PARP-1 inhibitors to rats was cardioprotective in postischemic reperfusion, reducing the size of myocardial infarction [55,56]. However, these studies were conducted before parthanatos were fully described, and other markers of this type of death were not analyzed.

PARP-1 inhibitors have been used in phase 1 clinical trials in humans with ST-Segment elevation myocardial infarction. The drug was well tolerated and reduced inflammatory markers and PARP activity > 90% [57].

Additionally, PARP-1 expression was studied in an animal model of pressure overload leading to HFrEF. It is upregulated in mice with aortic banding compared to wild-type mice [57].

It was also found that inhibition of PARP-1 contributed to the preservation of contractile function and reduced myocardial hypertrophy and fibrosis in mice with aortic banding [58].

Barany et al. [59] described a positive correlation between parthanatos markers (PARP activation, AIF translocation) in the blood of patients diagnosed with chronic HF. The study was conducted on a small sample, and most of the results were not statistically significant. However, the results suggest a role for this type of cell death in chronic HF.

That suggests that inhibition of parthanatos pathways could be a potential therapeutic target. This type of cell death has never been studied for HFpEF. The results of the above studies also cannot be unambiguously interpreted as real parthanatos because, in most of them, only PARP-1 was analyzed, which may accompany other types of PCD. Other features of this type of death were not studied in all of them.

#### 3.2.4. Pyroptosis

**Figure 5:**
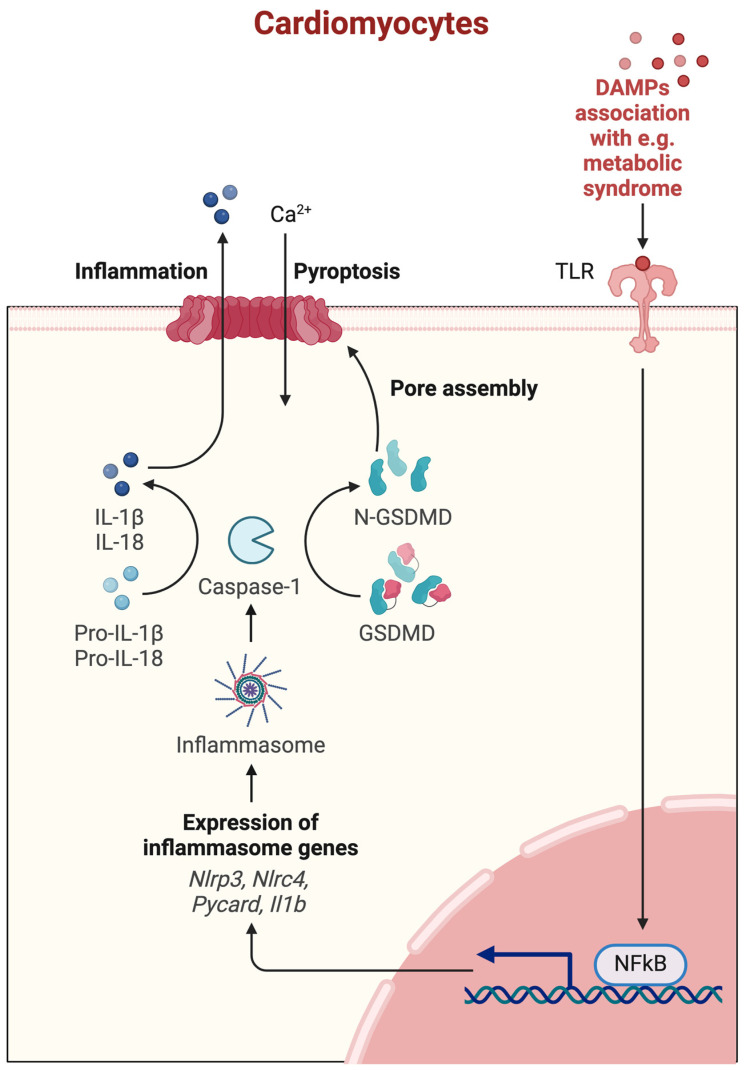
Molecular mechanism of pyroptosis. Ca^2+^—calcium ions, DAMPs—Danger/Damage Associated Molecular Patterns, TLR—toll-like receptor, GSDMD—gasdermin D, N-GSDMD—N-terminal domain of gasdermin D, IL—interleukin, pro-IL—pro-interleukin, NLRP3—NLR family pyrin domain containing 3, NLRC4—NLR family CARD domain-containing 4, Pycard—PYD and CARD domain containing, Il1b—interleukin 1b gene, NF-κB—nuclear factor kappa-light-chain-enhancer of activated B cells. Created with Biorender.com.

Pyroptosis is a form of PCD that is initiated in response to certain inflammatory signals, particularly those associated with infection and cellular damage (Figure 5) [2].

Some studies show that there might be a correlation between pyroptosis and the development of HFpEF, but mostly in the metabolic inflammation model of HFpEF (developed as a result of HFD). Xia et al. [60] proved that epicardial adipose tissue (EAT) through pyroptosis activation mediated by inflammasomes is one of the key sources of cardiovascular inflammation involved in the development of HFpEF. In a mouse model of HFpEF, established by the two-hit protocol with HFD and L-NAME, significantly increased production of IL-1β, IL-18, and inflammasomes was observed in EAT, which may be a consequence of the pyroptosis activation. Increased levels of other pyroptosis-related proteins were also noted: caspase-1 and gasdermin D (GSDMD). In addition, the expression of inflammasome NLR family pyrin domain containing 3 (NLRP3), the main mediator of pyroptosis, was higher in mice with the HFpEF phenotype. The results suggest that the regulation of pyroptosis significantly takes place in the EAT in mice with HFpEF. According to the authors, treatment targeting adipocyte-derived inflammation in EAT may be effective in HFpEF with a pro-inflammatory-metabolic phenotype. HFpEF is a heterogeneous entity, but other HFpEF phenotypes, often difficult to distinguish, are not characterized by such a significant metabolic component (and developed EAT). In addition to the obese and metabolic phenotype, the phenotype of vascular aging or the phenotype of a young patient with a low brain natriuretic peptide (BNP) level has also been distinguished; no studies have been conducted so far that would give results that could suggest an association with the ongoing pyroptosis process [61].

Koepp et al. [62] noted that obese patients with HFpEF have an increase in EAT mass and thickness, which may be a source of pro-inflammatory cytokines implicated in the progression of myocardial dysfunction. The association of the size of EAT indexed against body surface area and the development of HFpEF was also confirmed by Mahabadi et al. [63] among patients with initially present coronary artery disease.

While more research is needed to fully understand the role of pyroptosis in HF, these studies suggest that pyroptosis may be a potential therapeutic target for the treatment of HFpEF.

#### 3.2.5. NETosis

NETosis is a process in which oxidative stress-mediated neutrophil extracellular traps (NETs) are generated and accompanied by cell death. It has been proven to be induced by ROS derived primarily from reduced nicotinamide adenine dinucleotide phosphate (NADPH) oxidase-dependent activation of Ca^2+^ concentration in the cytoplasm and root-filled teeth (RFT) produced in the mitochondria. ROS activate myeloperoxidase (MPO), neutrophil elastase, and protein-arginine deiminase type 4 (PAD4), resulting in chromatin decondensation.

The activation of NETosis is a complex process that can be triggered by a variety of signals, including microbial components, cytokines, and chemical stimuli. Neutrophils recognize the pathogen through pattern recognition receptors, such as toll-like receptors and NOD-like receptors, which detect microbial components. When activated, neutrophils release NETs, which are composed of DNA, histones, and cytoplasmic proteins, forming a web-like structure that can trap various molecules, such as cytokines and chemokines, leading to increased inflammation [2].

##### NETs Induce Aseptic Inflammatory Process Associated with HFpEF

Ling and Xu [64] suggested that in HFpEF, the aseptic inflammatory response associated with NETs is integral to the process of myocardial damage, fibrosis, and remodeling. This conclusion was made based on previous research confirming the involvement of NETs in sterile inflammation [65] and the fact that modification of genes encoding proteins involved in NETosis caused specific structural changes in the heart, described later in the paper. Previous reports by Hage et al. [66] indicate that the inflammation and endothelial dysfunction present in HFpEF are potentiated by MPO, which concentration in plasma was higher in patients with HFpEF (N-terminal prohormone of brain natriuretic peptide > 300 ng/L, and an LVEF ≥ 45%) compared to healthy patients. MPO in complex with DNA can form NET in the tissue of a failing heart whose LVEF is preserved. Bolarkar et al. [67] demonstrated the association of neutrophil-to-leukocyte ratio with admission and its mortality among patients with acute HFpEF. The cohort consisted of 443 patients with acute HFpEF. During a follow-up of 0.3 to 4.9 years, 121 patients died. Both neutrophil-to-leukocyte ratios on admission and at discharge were significantly associated with mortality. This again suggests the involvement of neutrophils in worsening the course of HFpEF but does not prove a direct effect of NETosis. Although the association of some of the NET components with the development of HF (both HFrEF and HFpEF) has been suggested in many papers, the direct role of NETosis has not been proven [64].

##### Seipin Knockout Leads to NET Formation, Interstitial Fibrosis, and Ventricular Stiffness

Of the authors’ attempts to date involving NET-related gene manipulation, only one has unequivocally yielded clinical signs and features of HFpEF, but only in mouse models.

Seipin (also known as BSLC2) is a protein that plays a critical role in the regulation of lipid metabolism and adipocyte differentiation. It is predominantly expressed in adipose tissue, and mutations in the gene that encodes seipin are associated with severe congenital lipodystrophy [68]. It was previously shown that mice with seipin knockout develop HFpEF. It turned out that Seipin/Bslc2-deficient mice developed hearts with NET structures secreting MPO and histone-modified citrullinated DNA strands. Moreover, reactive interstitial fibrosis was observed, leading to ventricular stiffness [69].

##### Further Attempts: Modifying the Potent Signal Paths of NETosis in HF Mouse Models

Other modifications may suggest a link to the development of HFpEF, but this has not been clearly confirmed.

MPO is an enzyme that is found in the azurophilic granules of neutrophils and is involved in the production of ROS during NETosis. ROS helps to promote chromatin decondensation and the formation of NETs [70]. Higher levels of MPO were observed in patients with HFpEF compared to healthy samples [66]. In turn, ROS-related oxidative stress and inflammation lead to impaired systolic and diastolic cardiac function, hypertrophy, and remodeling [71].

According to Weckbach et al. [72], the 13-kD cytokine midkine is essential for the adhesion of polymorphonuclear neutrophils and their subsequent movement from blood vessels into surrounding tissues during acute inflammation. Moreover, it has been found to facilitate the recruitment of immune cells during chronic inflammation. Targeting the midkine gene resulted in inhibition of NETosis, neutrophil infiltration, and decreased fibrosis in mice with myosin-induced autoimmune myocarditis. In the same study, NETs were identified in patients with HF and proved to be inducted by midkine. However, only one among 14 patients was diagnosed with HFpEF [73].

Martinod et al. [74] demonstrated that deletion of the PAD4 gene in mice resulted in reduced NET formation and less perivascular fibrosis following induced contraction of the ascending aorta compared to wild-type mice. Mice that were either wild-type or deficient in PAD4 were subjected to aortic constriction surgery or sham surgery. The cellular composition of the left ventricle of the heart was analyzed using flow cytometry. The number of NETs/million cells was higher in mice without gene deletion. Moreover, longer follow-up showed that older mice with PAD4 gene deletion presented a better mean LVEF compared to wild-type mice. No significant changes were observed in structural parameters such as the diameter of the interventricular septum, the left ventricular posterior wall, and the left ventricular inner diameter.

Interleukin 8 receptor beta (CXCR2) plays a critical role in regulating neutrophil migration and recruitment to sites of infection or injury. During NETosis, CXCR2 is activated by certain chemokines, which triggers downstream signaling pathways that lead to the release of NETs [75]. Deletion of the CXCR2 gene resulted in reduced fibrosis within the myocardium in mice with HF (LVEF = 44.47 ± 12.68; some characterized by HFpEF but without exact figures provided by the authors) induced by Ang II at a dose of 1000 ng/kg/min [76].

It is possible that the RIPK3-MLKL (RIPK3—receptor-interacting protein kinase 3, MLKL—mixed lineage kinase domain-like) signaling pathway plays a role in the regulation of NETosis during inflammatory processes. It has been shown that the depletion of the RIPK3 gene leads to reduced DNA release in mice RIPK3-/- compared to RIPK3+/+ following monosodium urate crystal-induced NET [77]. However, other reports indicate that NET formation occurs without the involvement of the RIPK3 and MLKL signaling pathway and that this pathway is associated only with necroptosis [78].

#### 3.2.6. Necroptosis

Necroptosis can be triggered by the activation of certain receptors on the surface of cells, such as tumor necrosis factor (TNF) receptors or toll-like receptors, which can activate a series of signaling pathways. The activation of RIPK3 is the crucial event that triggers necroptosis in the death receptor pathway. After being activated, RIPK3 phosphorylates and activates MLKL, a pseudokinase, which then makes the cell membrane more permeable and oligomerizes it, ultimately causing necroptosis. The signaling pathway from RIPK3 to MLKL is the standard and most recognized way of initiating necroptosis [2].

##### The Role of RIPK3-MLKL Pathway in HFpEF

Necroptosis is a newly discovered cell death pathway that relies on the activity of RIPK3 and MLKL protein. Studies using RIPK3 and MLKL knockout mice have shown that necroptosis triggers inflammation, and recent research suggests that RIPK3 may contribute to the production of pro-inflammatory cytokines independently of necroptosis [78]. RIPK3 genetic ablation results in life extension in mice with HF.

Although previous reports include positive effects of RIPK3 genetic ablation in the post-infarction model of HF, the magnitude of the effect should be mentioned, and it cannot be ruled out whether it will be used in the treatment of HFpEF. In mice with the RIPK3 deletion, reductions in mRNA levels of the pro-inflammatory cytokines TNF-α and IL-6 and ROS in the heart tissue were observed, among other things. This may be a potential target for reducing the severity of inflammatory processes in HFpEF [79].

##### Caspase-8 Is Responsible for the Balance between Apoptosis and Necroptosis

Caspase-8 activates apoptosis and inhibits necroptosis by cleaving and inactivating key molecules in the necroptotic pathway. Its regulation is crucial in maintaining the balance between these two pathways to prevent excessive cell death and tissue damage.

##### Necroptosis Plays an Important Role in HFpEF

Previous studies confirm that necroptosis has significant effects on cardiovascular disease, but none have directly evaluated a model of HFpEF [80].

Cao et al. [81] confirmed the presence of severe necroptosis in HF induced by transverse aortic constriction. This type of procedure leads to HF with different phenotypes, including the concentric hypertrophy characteristic of HFpEF [82]. Wild-type mice, after transverse aortic constriction, showed increased expression of RIPK1, RIPK3, and MLKL compared to placebo mice. LVEF impairment was greater in RIPK3-knockout mice after transverse aortic constriction compared to RIPK3-/-mice after the same intervention. Exhaustion or inhibition of RIPK3 could reduce damage to the heart muscle, improve cardiac function, and weaken necroptosis, inflammatory response, and oxidative stress in mice with HF. However, these observations cannot be transferred to HFpEF without measurement of myocardial parameters (LVEF, diastolic dysfunction), and these were not provided by the authors.

Song et al. [83], in a mouse model, showed that SIRT3 expression in the myocardium of animals with diabetic cardiomyopathy induced by streptozocin injections was lower compared to healthy animals. To further explore the role of the gene, SIRT3-knockout mice were again injected with streptozocin. LVEF after 12 weeks was lower in SIRT3-knockout mice compared to wild-type mice, but its mean value in all groups was higher than 40%, which may indicate an HFpEF phenotype, although the authors did not provide exact measurement values. Increased cardiac dysfunction and the amount of ROS were observed in SIRT3-deficient mice. Moreover, both in vitro and in vivo, SIRT3 deficiency increased the expression of necroptosis-related proteins, including but not limited to RIPK1 and RIPK3. The findings indicate that SIRT3 could be a new molecular target for therapy but in order to make such a conclusion for HFpEF, studies with the determination of precise HF parameters are necessary.

Below we provide a summary of the substances that, according to the above studies, have the effect of modulating non-apoptotic PCD (Table 3).

## 4. Conclusions

The development of HFpEF is a manifestation of impaired diastolic function. While the exact mechanisms underlying HFpEF are not fully understood, emerging evidence suggests that cell death plays a significant role in its development.

PCD of cardiomyocytes, which can be triggered by various factors such as oxidative stress, mechanical stress, and inflammation, has been implicated in the development of HFpEF. Increased loss of cardiomyocytes through various forms of PCD can result in hypertrophy of the remaining cells and cardiac fibrosis following fibroblast proliferation and activation and increased deposition of extracellular matrix. These contribute to myocardial stiffening and impaired diastolic function. Furthermore, the proliferation of apoptosis-resistant fibroblasts leads to the abnormal overgrowth of the myocardial matrix and fibrosis.

It should be remembered that changes such as impaired diastolic function of the heart can also be caused by endothelial dysfunction, chronic inflammation, and impaired mitochondrial function.

Understanding the role of cell death in HFpEF is crucial for developing targeted therapeutic interventions. Strategies aimed at reducing cardiomyocyte cell death, inhibiting fibrosis, improving endothelial function, and mitigating inflammation may hold promise in preventing or treating HFpEF. Inhibition of PCD appears to be a previously unrecognized mechanism of action of some drugs used in HF treatment. The efficacy of this pharmacological approach and the results of studies in animal models give hope that new drugs with new targets in the PCD pathways may be introduced for the treatment of patients with HFpEF. While we await the results of clinical trials and reports from clinical practice, further research to better describe the pathways that lead the cell into and out of the PCD pathway may help to identify more molecules that can be used to treat patients with HF.

## Figures and Tables

**Table 1:** Summary of present and potential therapies targeting apoptosis in HFpEF. ↑—increase, ↓—decrease, n.d.—no data, Bax—Bcl-2-associated X protein, PARP—poly (ADP-ribose) polymerase, TUNEL—terminal deoxynucleotidyl transferase dUTP nick end labeling, Bcl-2—B cell lymphoma 2.

First Author	Treatment	Model	Number	Effect on Apoptosis	Effect on Fibrosis
Yang et al. [11]	empagliflozin	rats with cardiorenal syndrome	18	↓ mitochondrial-Bax, cleaved caspase-3, and cleaved PARP	↓ myocardial fibrosis
		H9C2 cardiomyoblast cell line	1.0 × 10^6^ cells	↓ apoptosis measured by flow cytometry	-
Yeh et al. [12]	sacubitril/valsartan	rats with cardiorenal syndrome	24	↓ mitochondrial-Bax, cleaved caspase-3, and cleaved PARP	↓ myocardial fibrosis
		H9C2 cardiomyoblast cell line	0.5 × 10^5^ cells	↓ apoptosis measured by flow cytometry	-
Liu et al. [13]	melatonin	mice on a high-fat diet	n.d.	↓ apoptosis measured by TUNEL, ↑ anty-apoptotic Bcl-2	no influence on fibrosis
		H9C2 cardiomyoblast cell line	n.d.	medium with melatonin has a protective effect against apoptosis measured by TUNEL	-
Matyas et al. [14]	vardenafil	Zucker diabetic fatty rats	30	↓ apoptosis measured by TUNEL and cleaved PARP	protective effects on myocardial fibrosis
Lin et al. [15]	irisin	mice with diabetic cardiomyopathy	n.d.	↓ cleaved caspase-3 and ↑ the Bcl-2/Bax ratio	↓ myocardial fibrosis
		H9C2 cardiomyoblast cell line	n.d.	↓ apoptosis measured by TUNEL and cleaved caspase-3	-
		neonatal rat cardiomyocyte isolation	n.d.	↓ apoptosis measured by TUNEL and cleaved caspase-3	-
Wang et al. [16]	physical activity	mice with diabetic cardiomyopathy	24	↓ apoptosis measured by TUNEL and ↑ the Bcl-2/Bax ratio	↓ myocardial fibrosis

**Table 2:** Summary of present and potential therapies targeting ferroptosis in HFpEF. ↑—increase, ↓—decrease, n.d.—no data, Fe^2+^—ferrous ion, MDA—malondialdehyde, TFR1—transferrin receptor, ACSL4—acyl-CoA synthetase long-chain family member 4, 4-HNE—4-hydroxy-trans-2-nonenal, NOX4—nicotinamide adenine dinucleotide phosphate oxidase 4, GSH—glutathione, xCT—cystine/glutamate antiporter SLC7A11, FTH1—ferritin heavy chain 1, L-NAME—Nω-nitrol-arginine methyl ester, GPX4—glutathione peroxidase 4, FSP-1—ferroptosis suppressor protein 1, Nrf2—nuclear factor erythroid 2-related factor 2, SIRT3—Sirtuin 3, ROS—reactive oxygen species, TGF-β1—transforming growth factor-β1.

First Author	Treatment	Model	Number	Effect on Ferroptosis	Effect on Fibrosis
Ma et al. [37]	canagliflozin	rats with a high-salt diet	36	↓ ferroptosis: ↓ Fe^2+^, MDA, TFR1, ACSL4, 4-HNE, NOX4 and ↑ GSH, xCT, FTH1	↓ myocardial fibrosis
Kitakata et al. [38]	imeglimin	mice with a high-fat diet and L-NAME	n.d.	↓ ferroptosis: ↑ GPX4	↓ myocardial fibrosis
Zhang et al. [39]	levosimendan	mice with a high-fat diet and L-NAME	n.d.	↓ ferroptosis: ↑ xCT, GPX4, FSP-1, ↓ NOX4, total iron content, Fe^2+^, MDA, 4-HNE	not studied
Zhang et al. [40]	elabela	mice with angiotensin II-induced hypertension	n.d.	↓ ferroptosis: ↑ GPX4, Nrf2, xCT	↓ myocardial fibrosis
	ferrostatin-1			↓ ferroptosis: ↑ GPX4, Nrf2, xCT	↓ myocardial fibrosis
Su et al. [42]	ferrostatin-1	mice with SIRT3 knockout (ferroptosis-dependent cardiac fibrosis)	n.d.	↓ ferroptosis: ↓ 4-HNE level, ROS formation, TGF-β1 expression	↓ myocardial fibrosis

**Table 3:** Summary of present and potential therapies targeting other PCD in HFpEF. ↑—increase, ↓—decrease, n.d.—no data, TUNEL—terminal deoxynucleotidyl transferase dUTP nick end labeling, GSDMD—gasdermin D, IL—interleukin, EAT—epicardial adipose tissue.

First Author	Treatment	Model	Number	Type of PCD	Effect on PCD	Effect on Fibrosis
Xie et al. [49]	metformin	OVE26 mice with diabetic cardiomyopathy	24	autophagy	↑ autophagy: measured by electron micrographic analysis of ventricular tissue double membrane-bound autophagic vesicles, ↑ LC3-II level, ↑ Beclin1 protein expression	not studied
				apoptosis	↓ apoptosis measured by TUNEL	
		cardiomyocyte-derived cell line HL-1	n.d.	autophagy	↑ autophagy: ↑ LC3-II level	-
Xia et al. [60]	Spironolactone and rosuvastatin	HFpEF mice with metabolic disorders	n.d.	pyroptosis	↓ pyroptosis: ↓ caspase-1, GSDMD, IL18 and IL-1β, NLRP3 expression in the EAT	not studied
				autophagy	↓ autophagy: ↓ LC3 level	

## Data Availability

No new data were created or analyzed in this study.
